# Association between companion animal ownership and overall life satisfaction in Seoul, Korea

**DOI:** 10.1371/journal.pone.0258034

**Published:** 2021-09-30

**Authors:** Jeehyun Kim, Byung Chul Chun

**Affiliations:** 1 Graduate School of Public Health, Korea University, Seoul, Republic of Korea; 2 Department of Preventive Medicine, Korea University College of Medicine, Seoul, Republic of Korea; 3 Department of Healthcare Sciences, Transdisciplinary Major in Learning Health Systems, Graduate School, Korea University, Seoul, Republic of Korea; Memorial University of Newfoundland, CANADA

## Abstract

This study aimed to analyze the association between companion animal ownership, the sub-factors of this ownership (the species and number of owned pets), and overall life satisfaction (OLS). Data was obtained from the publicly available responses to the 2017 Seoul Survey, conducted among Seoul-based Korean locals aged ≥ 15 years (N = 42,687; pet owners = 8,708, non-owners = 33,979). Propensity score was calculated by performing logistic regressions with covariates and data was matched using the nearest-neighbor method. Further, multiple linear regression was performed to analyze this association using the matched data. Additionally, survey-weighted multiple regressions were performed: 1) within pet owners, and 2) after stratifying owners with the number of pets owned. Pet owners in Seoul, South Korea reported higher levels of OLS than non-owners, even after controlling for covariates—age, sex, marital status, family size, family income, job, education, types of housing, housing tenure. Owners with both dogs and cats showed the highest average OLS scores (owners with 2 pets: Mean [M] = 58.05, Standard Deviation [SD] = 0.67; owners with ≥ 3 pets: M = 59.03, SD = 1.02), followed by single pet owners of either a cat (M = 56.64, SD = 0.37) or a dog (M = 56.14, SD = 0.13). Single pet owners reported significantly higher levels of OLS than those with 2 or ≥ 3 pets when pet types were adjusted for. When owners had a single pet, pet types (dog or cat) did not generate a significant difference in OLS scores. Among owners with 2 or ≥ 3 pets, however, owners with both dogs and cats had higher OLS scores than dog owners. This research has significant implications for promoting future study on companion animal effects for improving human health and well-being. Mechanisms of the effect, including cultural factors, should be further investigated.

## Introduction

Recent structural changes in the population due to aging, increasing single-person households, and lifestyle changes have subsequently led to a steady increase in pet-owning households and pet-related consumerism [1,2]. In South Korea, the proportion of pet-owning households was 26.4% (5.91 million households) in 2019 [3]; furthermore, the pet industry in South Korea is expected to grow from 1,544 billion won (₩) in 2018 to 3,498 billion won in 2027 [1]. Further, there is an increased interest in the effects of companion animal ownership on health and well-being to ensure a sound and healthy symbiosis of companion animals and their owners.

Companion animals share affection and friendship, help in relieving stress, and act as family members [2–8]. The “companion animal effect” states that companion animals are positively associated with the owners’ physical, mental, and social health [4]; this hypothesis has been supported by several existing studies [5–8]. According to a Chinese study, pet ownership had a positive causal effect on human health [9]. Additionally, a national representative longitudinal study in Germany and Australia revealed that owning a pet could reduce the number of visits to the doctor, even after minimizing confounding effects through the propensity score matching method [10].

However, contradictory findings suggest that companion animal ownership has little or no association with human health and well-being [11–15]. Moreover, some studies report several risks associated with companion animals, such as zoonosis, allergies, and biting [5]. In addition, pet-ownership was not associated with any statistically significant effects on children’s health, after adjusting for confounding factors [13]. Similarly, a study conducted in Finland reported that perceived health levels are negatively associated with pet-ownership [14]. Another study conducted on a representative sample of the entire Swedish population revealed that pet owners are more likely to experience mental health problems than non-owners [15].

Therefore, the positive association between companion animal ownership and human health and well-being is yet to be explored thoroughly [16], due to limitations in study samples that focus on sub-populations (i.e., older adults [2,11,16], children [13,17], or internet users [18]); study design (i.e., cross-sectional study [2,11,14,15,18–21]), small sample size [5], and lack of cultural diversity. The increased number of pet-owning households and the growing interest in pets in South Korea [3] has encouraged the media to emphasize the positive effects of companion animals on human health and well-being [22]; however, the existing research on this topic in South Korea is insufficient and lacks representative samples [19,20,23].

Previous studies have reported that sub-factors of pet ownership—the species and number of owned pets—were associated with human health and well-being [18–20,24,25]. However, current findings on this topic are inconclusive. A recent South African study revealed that dog owners experience higher life satisfaction than cat owners [18]. Another study reported that an improvement in minor health problem and health behavior lasts longer in dog owners compared to cat owners [24]. However, a New-Zealand study reported that life satisfaction is unrelated with pet type [25]. Some studies, which have small number of sample size, suggested that the number of pets owned had no significant effects on quality of life; physical, social, financial, and psychological satisfactions; and subjective well-being [19,20].

This study aimed to examine the association between companion animal ownership and overall life satisfaction, one measure of human well-being, through the following hypotheses: 1) Companion animal owners in Seoul, South Korea will have higher overall life satisfaction scores than non-owners, and 2) Companion animal owners’ levels of overall life satisfaction will differ based on the species and number of pets they own.

## Materials and methods

### Data

This study was conducted using the publicly available data obtained from the 2017 Seoul Survey [26]. This survey, conducted by the Seoul Metropolitan Government in 2017, gathered data both from both local Koreans and foreigners, but we used the data obtained from the Korean sample only. This sample included households and their members aged 15 years or older, who lived in Seoul in September 2017.

Since 2003, the Seoul Survey has been conducted to investigate 12 sectors in order to aid policy-making decisions, including: population and household, economy, housing and living sectors (asked every year); personal health and healthcare, safety and disaster, governance, Seoul welfare services, a set of values and social awareness sectors (asked alternate years); and education and childcare, environment, transportation, culture and leisure sectors (asked every second year in place of the second set of sectors). Companion animal-related items were added to the interview questionnaire in 2013 and asked every year. In 2017, this survey examined the sectors of personal health and healthcare, safety and disaster, governance, Seoul welfare services, and a set of values and social awareness.

A total of 20,000 households, with 42,687 people, were surveyed from September 1–30, 2017. The survey was conducted by a trained interviewer through face-to-face interviews. In the absence of an interviewee, the interviews were rescheduled and examined. Participants were recruited using stratified cluster sampling. Detailed information about the 2017 Seoul Survey methodology is available elsewhere [27].

### Variables

Companion animal ownership data were obtained from the 2017 Seoul Survey. The respondents were asked whether they owned companion animals (1) or not (0). The responses to “How many companion animals do you presently own: dog/cat/others?” were categorized to indicate the species and number of owned pets. The companion animal species were classified as “dogs (1; owning only dogs or owning both dogs and pets other than dogs and cats),” “cats (2; owning only cats or owning both cats and pets other than dogs and cats),” “dogs and cats (3; owning both dogs and cats or owning dogs, cats, and other pets),” and “others (4; owning pets other than dogs and cats).” Dummy variables were created by using the case of “dogs (1)” as the reference group. Similarly, number of pets owned was classified as follows: “one pet (1),” “two pets (2),” and “three or more pets (3).” Dummy variables were created using the case of “one pet (1)” as the reference group.

The 2017 Seoul survey enquired respondents’ degree of satisfaction with their: standard of living, life achievements, personal relationships, sense of safety, feeling of belongingness toward a community, future security, amount of time spent doing what they like, and local environment quality. Each item was scored from 0 (*not satisfied at all*) to 10 (*very satisfied*). The 2017 Seoul Survey evaluated overall life satisfaction by employing the domain evaluations module, which is recommended as a measure of subjective well-being by the Organization for Economic Co-operation and Development (OECD), across diverse life aspects based on the policy needs of each institute [28]. The questions incorporated in this module were derived mainly from the Personal Wellbeing Index-Adult (PWI-A), which showed adequate construct and convergent validity [29,30]. Additionally, experts reported sufficient content validity of the life satisfaction measures in the 2017 Seoul Survey [27]. The Cronbach’s alpha coefficient for the eight life satisfaction sections was 0.919. The overall life satisfaction was evaluated as the sum of the eight life satisfaction parameters. The overall life satisfaction scores ranged between 2 and 80, where higher scores indicated higher levels of overall life satisfaction.

Age, sex, marital status, family size, family income, job, education, types of housing, and housing tenure type were considered as the potential confounding factors, associated with overall life satisfaction, pet ownership, and sub-factors of ownership (the species and number of owned pet).

### Statistical analysis

Descriptive statistics and chi-squared tests were used to analyze the demographic characteristics of the population by companion animal ownership. Additionally, descriptive analysis, independent samples *t*-test, analysis of variance (ANOVA), and Scheffe’s test as a post-hoc analysis, were employed for examining the overall life satisfaction levels among the total population, the pet owners’ group, and the non-owners’ group. Moreover, descriptive analysis was performed for each combination of the two sub-factor variables—the species and number of pets owned.

Multiple linear regression analyses were performed using propensity score matching to evaluate the associations between companion animal ownership and overall life satisfaction [31,32]. Propensity score is a methodological adjustment, which, here, accounts for the differences in socio-demographic traits between pet owners and non-owners that could influence mental and physical health outcomes [33]. Propensity score—the probability that an individual owns a pet—was computed by performing logistic regression analyses including the following covariates: age, sex, marital status, family size, family income, job, education, types of housing, housing tenure. This study employed the nearest-neighbor method with a ratio of 1:2 (pet-owners to non-owners) and a caliper width of 0.01 to match the data. Shorter caliper distance gives stricter threshold for matching [32]. Thus, the matched data generated by this method only contained non-owners (control group) and owners (treatment group) who were matched. Further, we performed multiple linear regression analyses using the matched data to examine the associations between companion animal ownership and overall life satisfaction. Significant differences in overall life satisfaction (outcome) due to pet-ownership may indicate a causal impact of pet-ownership on the outcome variable, because of the reduction in unobserved heterogeneity through matched data.

Additionally, survey-weighted multiple linear regression analyses were performed to analyze the associations between the sub-factors of ownership and overall life satisfaction among the pet owners’ group. Propensity score matching was not conducted among pet owners because of the small number of whom both owned dog and cat (n = 265, 3.0%) and whom owned three or more pets (n = 191, 2.2%). However, while examining this group, we excluded the “others (4)” category from the species of pets owned to clarify the differences between each group of species, which are “dogs (1),” “cats (2),” and “dogs and cats (3)”. Moreover, in order to clarify the effect of the species owned, we stratified each study subject with the number of pets owned and conducted survey-weighted multiple linear regression considering the interaction between the species and the number of pets owned.

All data were statistically analyzed using SPSS version 19 (IBM), and R version 4.0.3. Statistical significance was set at p < 0.05, and multicollinearity was examined using the Variance Inflation Factor (VIF < 4). This study was exempted from ethical approval by the Institutional Review Board (IRB) of Korea University (IRB exemption number: KUIRB-2018-0079-01).

## Results

Table 1 presents the demographic characteristics of the study participants (N = 42,687). Of these, 8,708 participants (20.4%) owned companion animals. The chi-squared test results showed that age, sex, marital status, family size, family income, education, types of housing, housing tenure type (all p < 0.001), and job (p = 0.013), differed significantly by ownership status.

**Table 1:** Demographic characteristics of participants by pet ownership.

	Total		Owners		Non-owners		*p*-value^a^
	N	%	N	%	N	%	
**Total**	42,687	100	8,708	20.4	33,979	79.6	
**Age (years)**							< 0.001
**15 to 19**	2,473	5.8	595	24.1	1,878	75.9	
**20 to 29**	7,054	16.5	1,644	23.3	5,410	76.7	
**30 to 39**	7,813	18.3	1,296	16.6	6,517	83.4	
**40 to 49**	8,040	18.8	1,606	20.0	6,434	80.0	
**50 to 59**	7,544	17.7	1,751	23.2	5,793	76.8	
**60 to 69**	5,814	13.6	1,211	20.8	4,603	79.2	
**70 to 79**	3,052	7.2	488	16.0	2,564	84.0	
**≥ 80**	896	2.1	117	13.1	779	86.9	
**Sex**							< 0.001
**Female**	21,889	51.3	4,611	21.1	17,278	78.9	
**Male**	20,798	48.7	4,097	19.7	16,701	80.3	
**Marital status**							< 0.001
**Married or cohabited**	24,856	58.2	4,939	19.9	19,917	80.1	
**Single**	12,952	30.3	2,849	22.0	10,103	78.0	
**Divorced**	1,952	4.6	401	20.5	1,551	79.5	
**Bereaved**	2,927	6.9	520	17.8	2,407	82.2	
**Family size**							< 0.001
**1**	6,365	14.9	1,173	18.4	5,192	81.6	
**2**	9,664	22.6	2,047	21.2	7,617	78.8	
**3**	11,410	26.7	2,311	20.3	9,099	79.7	
**4**	11,210	26.3	2,337	20.8	8,873	79.2	
**≥ 5**	4,039	9.5	841	20.8	3,198	79.2	
**Family income (₩)**							< 0.001
**< 1,000,000**	1,750	4.1	252	14.4	1,498	85.6	
**1,000,000 to 1,999,999**	3,749	8.8	603	16.1	3,146	83.9	
**2,000,000 to 2,999,999**	5,798	13.6	1,039	17.9	4,759	82.1	
**3,000,000 to 3,999,999**	7,662	17.9	1,319	17.2	6,343	82.8	
**4,000,000 to 4,999,999**	8,117	19.0	1,841	22.7	6,276	77.3	
**≥ 5,000,000**	15,612	36.6	3,655	23.4	11,957	76.6	
**Job**							0.013
**Management profession**	2,464	5.8	560	22.7	1,904	77.3	
**White collar**	14,078	33.0	2,830	20.1	11,248	79.9	
**Blue collar**	9,057	21.2	1,882	20.8	7,174	79.2	
**Others**	17,088	40.0	3,436	20.1	13,652	79.9	
**Education**							< 0.001
**≤ Middle school**	7,062	16.5	1,323	18.7	5,739	81.3	
**High school**	15,342	35.9	3,252	21.2	12,090	78.8	
**≥ College degree**	20,283	47.5	4,133	20.4	16,150	79.6	
**Types of housing**							< 0.001
**Detached house**	13,199	30.9	2,817	21.3	10,383	78.7	
**Apartment**	20,050	47.0	4,217	21.0	15,833	79.0	
**Others**	9,438	22.1	1,675	17.7	7,762	82.3	
**Housing tenure type**							< 0.001
**Private**	21,585	50.6	4,733	21.9	16,853	78.1	
**Lease**	11,351	26.6	2,148	18.9	9,204	81.1	
**Others**	9,751	22.8	1,828	18.7	7,922	81.3	
**Species**							-
**Dogs**	-	-	7,464	85.7	-	-	
**Cats**	-	-	924	10.6	-	-	
**Dogs and cats**	-	-	265	3.0	-	-	
**Others**	-	-	55	0.6	-	-	
**Number of pets owned**							-
**1**	-	-	7,301	83.8	-	-	
**2**	-	-	1,216	14.0	-	-	
**≥ 3**	-	-	191	2.2	-	-	

^a^*p*-values were calculated with chi-square tests.

Among the owners’ group, a total of 7,464 participants (85.7%) were dog owners, followed by 924 cat owners (10.6%), 265 owners of both dogs and cats (3.0%), and 55 other pet owners (0.6%). With respect to the number of pets owned, 7,301 participants (83.8%) owned only one animal, 1,216 (14.0%) owned two animals, and 191 (2.2%) owned three or more animals.

Table 2 presents the results of descriptive analysis, independent samples *t*-test, and ANOVA on overall life satisfaction within total population, owners’ group, and non-owners’ group. Overall life satisfaction scores of the total population ranged from 2–80. Owners (Mean [M] = 56.02, Standard Deviation [SD] = 10.25) displayed higher average overall life satisfaction levels than non-owners (M = 54.79, SD = 10.68).

**Table 2:** Descriptive statistics, t-test, and ANOVA on overall life satisfaction by pet ownership.

		**Total population**			**Owners**			**Non-owners**		
		**Mean**	**SD**	*p*-value*	**Mean**	**SD**	*p*-value*	**Mean**	**SD**	*p*-value*
**Ownership status**										
**Owners**		56.02	10.25	< 0.001	-	-	-	-	-	-
**Non-owners**		54.79	10.68		-	-		-	-	
**Age (years)**										
**15 to 19**		56.82^f^	9.53	< 0.001	58.28^e^	9.16	< 0.001	56.36^e,f^	9.60	< 0.001
**20 to 29**		56.26^e,f^	9.70		56.56^c,d,e^	9.48		56.17^e,f^	9.77	
**30 to 39**		56.89^f^	9.78		57.69^d,e^	9.61		56.73^f^	9.81	
**40 to 49**		55.83^d,e^	10.02		56.90^c,d,e^	9.67		55.56^d,e^	10.09	
**50 to 59**		54.98^d^	10.66		55.78^c,d^	10.28		54.74^d^	10.76	
**60 to 69**		53.35^c^	11.15		54.69^c^	10.63		53.00^c^	11.26	
**70 to 79**		50.03^b^	11.66		50.79^b^	11.75		49.88^b^	11.64	
**≥ 80**		46.09^a^	12.79		45.84^a^	13.69		46.13^a^	12.66	
**Sex**										
**Female**		54.76	10.73	< 0.001	55.78	10.32	0.018	54.49	10.82	< 0.001
**Male**		55.34	10.46		56.30	10.16		55.11	10.52	
**Marital status**										
**Married or cohabited**		55.79^c^	9.98	< 0.001	56.47^c^	9.87	< 0.001	55.63^c^	10.00	< 0.001
**Single**		55.82^c^	10.19		57.08^c^	9.58		55.47^c^	10.33	
**Divorced**		49.76^b^	13.06		51.20^b^	11.93		49.39^b^	13.32	
**Bereaved**		48.75^a^	12.45		49.79^a^	12.48		48.53^a^	12.43	
**Family size**										
**1**		52.32^a^	12.39	< 0.001	54.19^a^	11.15	< 0.001	51.89^a^	12.62	< 0.001
**2**		54.13^b^	10.82		55.54^b,c^	10.77		53.75 ^b^	10.80	
**3**		55.84^c,d^	10.04		56.44^c,d^	9.88		55.69^c^	10.08	
**4**		56.33^d^	9.76		57.34^d^	9.37		56.06^c^	9.85	
**≥ 5**		55.72^c^	9.83		54.95^a,b^	10.38		55.92 ^c^	9.67	
**Family income (₩)**										
**< 1,000,000**		44.38^a^	14.15	< 0.001	46.02^a^	13.31	< 0.001	44.11 ^a^	14.27	< 0.001
**1,000,000 to 1,999,999**		49.66^b^	11.98		50.78^b^	11.66		49.45^b^	12.03	
**2,000,000 to 2,999,999**		53.59^c^	10.92		54.46^c^	10.66		53.40 ^c^	10.97	
**3,000,000 to 3,999,999**		55.26^d^	10.00		55.80^c,d^	9.99		55.15^d^	9.99	
**4,000,000 to 4,999,999**		56.76^e^	9.39		57.51^d^	9.30		56.54 ^e^	9.41	
**≥ 5,000,000**		57.08^e^	9.24		57.36^d^	9.42		56.99^e^	9.18	
**Job**										
**Management profession**		56.49^b^	10.02	< 0.001	56.65^b^	10.32	< 0.001	56.45^b^	9.94	< 0.001
**White collar**		56.99^b^	9.40		57.46^b^	9.19		56.88 ^b^	9.45	
**Blue collar**		53.69^a^	10.83		54.76^a^	10.46		53.41 ^a^	10.91	
**Others**		53.95^a^	11.22		55.43^a^	10.78		53.57 ^a^	11.30	
**Education**										
**≤ Middle school**		50.37^a^	12.24	< 0.001	51.78^a^	12.31	< 0.001	50.05 ^a^	12.20	< 0.001
**High school**		54.71^b^	10.38		55.64^b^	9.94		54.46 ^b^	10.48	
**≥ College degree**		56.92^c^	9.58		57.68^c^	9.29		56.73 ^c^	9.65	
**Types of housing**										
**Detached house**		54.11^a^	10.95	< 0.001	54.77^a^	10.57	< 0.001	53.94 ^a^	11.04	< 0.001
**Apartment**		55.78^c^	10.21		56.87^b^	9.95		55.49 ^b^	10.25	
**Others**		54.79^b^	10.83		56.00^c^	10.21		54.53 ^c^	10.94	
**Housing tenure type**										
**2Private**		56.05^c^	9.60	< 0.001	56.78^c^	9.42	< 0.001	55.85 ^c^	9.64	< 0.001
**Lease**		55.5^b^	10.17		55.67	10.43		55.49^b^	10.11	
**Others**		52.26^a^	12.55		54.48^a^	11.78		51.74 ^a^	12.67	
**Species**	**Number of pets owned**									
**Dogs**	**1**				56.14^a,b,c^	0.13	< 0.001			
	**2**				54.86^a,b,c^	0.36				
	**≥ 3**				51.94^a^	1.23				
**Cats**	**1**				56.64^a,b,c^	0.37				
	**2**				53.84^a,b^	0.82				
	**≥ 3**				54.65^a,b,c^	1.64				
**Dogs and cats**	**2**				58.05^b,c^	0.67				
	**≥ 3**				59.07^c^	1.02				

SD: The standard difference for mean.

*p-values were calculated with independent sample *t*-tests or ANOVA.

^a,b,c,d,e,f^ Same letter indicates statistically non-significant differences based by Scheffe’s post-hoc analysis.

In the overall population, males, singles, white-collar workers, and those having higher education, living in an apartment, and owning a house showed higher levels of overall life satisfaction. Additionally, overall life satisfaction generally decreased with age, while it increased with growth in household income. However, single-member households displayed the lowest average overall life satisfaction.

Age, sex, marital status, family size, family income, job, education, type of housing, and housing tenure type were associated with significant differences in the overall life satisfaction levels among the pet owners’ group and among non-owners’ group (all p <0.05).

Average overall life satisfaction among dog owners decreased with an increase in the number of pets owned (single pet owners: M = 56.14, SD = 0.13). Similarly, among cat owners, single cat owner reported the highest overall life satisfaction levels (M = 56.64, SD = 0.37). However, among owners who raised both dogs and cats, those with three or more pets reported higher overall life satisfaction levels (M = 59.07, SD = 1.02) than those with two pets (M = 58.05, SD = 0.67). In other words, owners of both dogs and cats showed the highest average overall life satisfaction levels, followed by single pet owners of either a cat or a dog.

Propensity score matching reduced the imbalance of covariates (S1 Table). The matched data comprised 8,654 pet-owners and 17,293 non-owners. Table 3 presents the results of the multiple linear regression analyses conducted using matched data to identify the association between pet ownership and overall life satisfaction.

**Table 3:** Multiple linear regression on overall life satisfaction using propensity score matching.

	Coefficient	SE	95% CI
**Ownership status**			
**Owners**	0.510	0.126	0.263–0.757
**Non-owners**	ref.		
**Age (years)**	-0.632	0.057	-0.745 –-0.520
**Sex**			
**Female**	ref.		
**Male**	0.342	0.128	0.090–0.594
**Marital status**			
**Married or cohabited**	ref.		
**Single**	-1.339	0.202	-1.735 –-0.943
**Divorced**	-3.100	0.329	-3.745 –-2.456
**Bereaved**	-2.463	0.277	-3.007 –-1.919
**Family size**	-0.722	0.072	-0.863 –-0.581
**Family income (₩)**	1.115	0.057	1.004–1.226
**Job**			
**Management profession**	-0.815	0.263	-1.330 –-0.300
**White collar**	ref.		
**Blue collar**	-1.009	0.174	-1.35 –-0.668
**Others**	0.090	0.169	-0.241–0.421
**Education**			
**≤ Middle school**	-2.867	0.214	-3.286 –-2.449
**High school**	-0.918	0.149	-1.21 –-0.627
**≥ College degree**	ref.		
**Types of housing**			
**Detached house**	-0.686	0.137	-0.954 –-0.418
**Apartment**	ref.		
**Others**	-0.331	0.162	-0.649 –-0.012
**House tenure type**			
**Private**	ref.		
**Lease**	-1.051	0.143	-1.331 –-0.771
**Others**	-2.850	0.245	-3.329 –-2.371

SE: The standard error for coefficient; 95% CI: 95% Confidence Interval.

The model indicated no multicollinearity among the independent variables (VIF < 4). Pet ownership status (Coefficient = 0.510, 95% Confidence Interval [CI] = 0.263–0.757) was found to be positively associated with overall life satisfaction, even after accounting for the unobserved heterogeneity between owners and non-owners. Thus, pet owners reported higher overall life satisfaction levels than non-owners.

Table 4 presents the results of survey-weighted multiple linear regression analysis among the owners’ group, as stratified by the number of pets owned. The overall life satisfaction level among owners with only one pet was 1.455 points higher (95% CI = -2.288 –-0.628) than those with two pets, and 2.279 points higher than those with three or more pets (95% CI = -3.940 –-0.618) under controlled conditions. Thus, overall life satisfaction decreased as the number of pets increased when pet types were adjusted for.

**Table 4:** Survey-weighted multiple linear regression on overall life satisfaction by number of pets owned.

	Owners (N = 8,653)			Number of pets owned = 1 (N = 7,265)			Number of pets owned = 2 (N = 1,211)			Number of pets owned ≥ 3 (N = 177)		
	Coefficient	SE	95% CI	Coefficient	SE	95% CI	Coefficient	SE	95% CI	Coefficient	SE	95% CI
**Number of pets owned**												
**1**	ref.			-	-	-	-	-	-	-	-	-
**2**	-1.455	0.425	-2.288 –-0.623	-	-	-	-	-	-	-	-	-
**≥ 3**	-2.279	0.847	-3.940 –-0.618	-	-	-	-	-	-	-	-	-
**Species**												
**Dog**	ref.			ref.			ref.			ref.		
**Cats**	0.053	0.440	-0.809–0.916	0.315	0.475	-0.617–1.247	-0.932	1.043	-2.976–1.113	-0.151	2.271	-4.601–4.300
**Dogs and cat**	4.051	0.740	2.600–5.502	-	-	-	2.449	0.835	0.812–4.087	5.923	1.244	3.484–8.362
**Age (years)**	-0.737	0.143	-1.018 –-0.456	-0.665	0.158	-0.975 –-0.355	-1.267	0.347	-1.947 –-0.587	-0.749	0.483	-1.696–0.197
**Sex**												
**Female**	ref.			ref.			ref.			ref.		
**Male**	-0.022	0.284	-0.579–0.534	-0.021	0.308	-0.625–0.583	0.067	0.713	-1.330–1.465	2.721	1.370	0.036–5.406
**Marital Status**												
**Married of cohabited**	ref.			ref.			ref.			ref.		
**Single**	-0.903	0.457	-1.799 –-0.007	-0.899	0.503	-1.885–0.087	-1.674	1.118	-3.865–0.516	4.022	1.769	0.554–7.490
**Divorced**	-4.262	0.837	-5.902 –-2.621	-4.223	0.915	-6.017 –-2.429	-2.787	1.924	-6.558–0.984	-7.695	2.760	-13.105 –-2.286
**Bereaved**	-3.232	0.780	-4.760 –-1.704	-3.212	0.885	-4.947 –-1.477	-4.005	1.407	-6.761 –-1.248	4.547	2.472	-0.299–9.393
**Family size**	-1.099	0.177	-1.446 –-0.751	-0.792	0.184	-1.153 –-0.43	-2.521	0.420	-3.345 –-1.698	-0.104	0.642	-1.361–1.154
**Family income (₩)**	1.240	0.138	0.970–1.511	1.188	0.147	0.900–1.477	1.260	0.332	0.610–1.910	2.312	0.611	1.115–3.510
**Job**												
**Management profession**	-1.214	0.578	-2.346 –-0.081	-1.456	0.643	-2.717 –-0.196	-0.082	1.326	-2.682–2.518	-0.350	2.216	-4.693–3.993
**White collar**	ref.			ref.			ref.			ref.		
**Blue collar**	-0.699	0.377	-1.437–0.039	-0.297	0.413	-1.106–0.513	-2.409	0.876	-4.125 –-0.692	-3.268	2.532	-8.230–1.694
**Others**	0.276	0.362	-0.433–0.985	0.379	0.394	-0.393–1.151	-0.411	0.899	-2.172–1.350	0.174	1.868	-3.487–3.835
**Education**												
**≤ Middle school**	-3.626	0.518	-4.642 –-2.610	-3.752	0.555	-4.84 –-2.664	-2.286	1.439	-5.105–0.534	-4.903	2.108	-9.035 –-0.771
**High school**	-1.060	0.322	-1.691 –-0.429	-0.806	0.354	-1.499 –-0.113	-2.029	0.808	-3.613 –-0.445	-3.713	1.855	-7.348 –-0.078
**≥ College degree**	ref.			ref.			ref.			ref.		
**Types of housing**												
**Detached house**	-1.082	0.311	-1.692 –-0.472	-1.135	0.346	-1.813 –-0.457	-0.467	0.700	-1.839–0.905	0.987	1.346	-1.651–3.626
**Apartment**	ref.			ref.			ref.			ref.		
**Others**	-0.247	0.372	-0.977–0.482	-0.269	0.410	-1.074–0.535	-0.857	0.849	-2.521–0.806	6.641	1.578	3.548–9.734
**Housing tenure type**												
**Private**	ref.			ref.			ref.			ref.		
**Lease**	-1.594	0.291	-2.164 –-1.025	-1.294	0.321	-1.923 –-0.666	-3.096	0.719	-4.505 –-1.687	-2.390	1.471	-5.272–0.493
**Others**	-2.034	0.542	-3.096 –-0.972	-1.441	0.595	-2.607 –-0.274	-4.290	1.218	-6.677 –-1.902	-2.974	2.435	-7.747–1.798

SE: The standard error for coefficient; 95% CI: 95% Confidence Interval.

Among single pet owners, the difference in overall life satisfaction scores was not significant between dog and cat owners (Coefficient = 0.315, 95% CI = -0.617–1.247). Only those owners with both dogs and cats had higher life satisfaction scores than dog owners among owners of two pets (Coefficient = 0.2449, 95% CI = 0.812–4.087) and three or more pets (Coefficient = 5.923. 95% CI = 3.484–8.362).

## Discussion

This study analyzed the associations between companion animal ownership, sub-factors of this ownership, and overall life satisfaction, using the data of local Seoul citizens obtained from the 2017 Seoul Survey. The results revealed that pet ownership had significant effects on generating higher levels of overall life satisfaction. Pet owners with both dogs and cats had the highest average overall life satisfaction scores, followed by single pet owners of either a cat or a dog.

Most of the companion animal owning participants in Seoul, South Korea had dogs (85.7%), while only 10.6% owners had cats. This finding is in contrast to the results of both a United States study [34] and New Zealand study [25], which showed that cats were the more common pet type. One reason that Koreans may have less interest in owing cats as pets could be the historical depiction of cats as wicked in Korea [35]. However, the reasons behind Seoul citizen having this unique pet type proportion still needs further investigation.

Companion animal ownership was associated with higher levels of overall life satisfaction. This finding is consistent with previous findings suggesting that pet owners display better well-being indices, higher self-esteem, and better exercise performance than non-owners [36]. However, this result was inconsistent with a New Zealand study which reported that pet ownership was not associated with life satisfaction [25]. The differences in the study findings could have been because of the different characteristics of the study samples. For instance, while the majority of participants reported that they had pets in New Zealand study, the majority of participants in our study reported not having pets. Also, pet owners had lower levels of education than non-owners in New Zealand-based study, while pet owners were more likely to have a higher level of education than non-owners in our study. The shorter history in Korea of keeping animals as companions, compared to that in western society [35], might also explain the difference. Furthermore, the cross-sectional study design of both studies could have set limits on comparisons between them. It is recommended that future studies that examine the effects of pet ownership in the population take into account the characteristics and cultural differences of the population using a longitudinal study design.

According to the results of this study’s survey-weighted multiple linear regression to examine the association between the number of pets owned and overall life satisfaction, single pet ownership was associated with higher levels of overall life satisfaction than ownership of two and three or more pets, when pet species were adjusted for. This finding was in line with existing studies showing that single pet owners experienced higher subjective well-being and average overall quality of life than owners with two or more pets [19,20]; however, previous findings were not statistically significant. Small sample size in previous studies might have effect on the statistical insignificance in previous studies.

Furthermore, the results of our survey-weighted multiple linear regression within single pet owners revealed that overall life satisfaction levels were not significantly different between dog and cat owners. This result was inconsistent with earlier findings indicating that mental health and minor health issue improvements lasted for about 10 months among dog owners, while no improvement continued among cat owners [24]. This inconsistency might result from the different inclusion criteria of each study and different population base. Specifically, although the previous study excluded people who had owned pets before [24], the current study could not exclude them as it did not have data regarding the history of pet ownership. Future studies should clarify the causal relationship between the species of pets owned and overall life satisfaction, in accordance with the history of pet ownership.

However, the results of our survey-weighted multiple linear regression among owners who had two or more pets revealed that owning both dogs and cats was likely to generate higher levels of overall life satisfaction than owning either dogs or cats. This result was inconsistent with a previous New Zealand-based study that suggested that pet types (cats only; dogs only; cats and dogs) did not have association with life satisfaction, psychological distress, and self-esteem [25]. This inconsistency could have arisen because pet ownership is stratified by pet types as well as the number of pets owned in our study, and the previous study categorized pet ownership only with the type of pet. Also, different pet type preferences with previous study, which our studies having more dog owners than cat owners unlike previous study, could cause the inconsistency. Therefore, it is recommended that future studies include both pet types and the number of pets owned and conduct on various population to deepen our understanding of the interplay between pet ownership and human well-being.

Our study also has several limitations. Firstly, the current study design (i.e., cross-sectional) could not identify causal relationships. However, despite this design limitation, we established the causal relationship between pet ownership and overall life satisfaction by employing the propensity score matching method. Future studies can mitigate this limitation by incorporating companion animal-related items to the existing epidemiological panel studies, rather than devising new prospective studies [37]. Secondly, future research should examine the causal inference between the sub-factors of pet ownership and overall life satisfaction, which could not be studied in the current research because the number of participants was too small for: 1) those who were both dog and cat owners (n = 265, 3.0%), and 2) those who owned three or more pets (n = 191, 2.2%). Thus, propensity score matching was not conducted among these pet owners. Thirdly, this study did not consider the duration and history of pet ownership as covariates or as exclusion criteria. Previous studies revealed that the duration of pet ownership affects human health and well-being [19,21]. Additionally, participants with a history of pet ownership were excluded from a previous study [24]; in another previous study it was possible to infer a causal relationship between pet ownership and human health by evaluating the ownership status both at present and five years prior [10]. However, the current survey data did not provide information regarding the duration and history of pet ownership. Lastly, despite the advantages of the large sample for achieving statistical significance, the implementation of stratified sampling and the propensity score matching made the sample size smaller and even permitted us to adjust for confounders properly. Nevertheless, our results provided valuable insights into the association between pet ownership and overall life satisfaction among local Seoul citizens in 2017.

Therefore, despite these limitations, this study is insightful; it advocates a One Health approach called Zooeyia, which suggests that interaction with an animal, especially a companion animal, could positively affect human health [38]. Furthermore, this study provides evidence regarding the relationship between the sub-factors of pet ownership—species and the number of pets owned—and overall life satisfaction. By examining the association between companion animal ownership and overall life satisfaction, this research could assist in promoting the future research of the companion animal effect on improving human health and well-being. Mechanisms of the effect, including cultural factors, also should be investigated.

## Supporting information

S1 Table Distribution of covariates by pet-ownership before and after matching. (DOCX)
