# Supplementary material for: Association between companion animal ownership and overall life satisfaction in Seoul, Korea
Source: PLoS One. 2021 Sep 30;16(9):e0258034. doi: 10.1371/journal.pone.0258034 (PMC8483320; doi:10.1371/journal.pone.0258034)
Supplement: S1 Table — (DOCX) [file pone.0258034.s001.docx]

**Supporting Information**

**Supplementary Table 1. Distribution of covariates by pet-ownership before and after matching.**

| **Variables** | | **Before propensity score matching** | | | | **After propensity score matching** | | | |
| --- | --- | --- | --- | --- | --- | --- | --- | --- | --- |
|  |  | **Observed values** | | ***p*-value^a^** | **Standardized difference** | **Observed values** | | ***p*-value^a^** | **Standardized difference** |
| **Covariates** | | **Control group (n=34,026)** | **Treated group (n=8,661)** |  |  | **Control group (n=17,293)** | **Treated group (n=8,654)** |  |  |
| Age |  | 4.34 (1.74) | 4.22 (1.70) | <0.001 | 6.620 | 4.23 (1.72) | 4.22 (1.70) | 0.867 | 0.221 |
| Sex |  | 16,293 (47.9) | 4,056 (46.8) | 0.082 | 2.110 | 8,140 (47.1) | 4,054 (46.8) | 0.741 | 0.452 |
| Marital status | Married or cohabited | 22,577 (66.4) | 5,567 (64.3) | <0.001 | 0.122 | 11,284 (65.3) | 5,566 (64.3) | 0.106 | 0.419 |
|  | Single | 7,729 (22.7) | 2,242 (25.9) |  |  | 4,283 (24.8) | 2,239 (25.9) |  |  |
|  | Divorced | 1,344 ( 3.9) | 341 ( 3.9) |  |  | 635 ( 3.7) | 339 ( 3.9) |  |  |
|  | Bereaved | 2,376 ( 7.0) | 511 ( 5.9) |  |  | 1,091 ( 6.3) | 510 ( 5.9) |  |  |
| Family size |  | 2.89 (1.07) | 2.93 (1.06) | 0.010 | 3.098 | 2.93 (1.04) | 2.93 (1.06) | 0.621 | 0.650 |
| Family income |  | 4.49 (1.48) | 4.77 (1.38) | <0.001 | 19.937 | 4.78 (1.36) | 4.77 (1.38) | 0.841 | 0.264 |
| Job | Management profession | 1,905 ( 5.6) | 563 ( 6.5) | <0.001 | 3.784 | 1,056 ( 6.1) | 562 ( 6.5) | 0.003 | 0.803 |
|  | White collar | 10,978 (32.3) | 2,768 (32.0) |  |  | 5,835 (33.7) | 2,766 (32.0) |  |  |
|  | Blue collar | 7,035 (20.7) | 1,931 (22.3) |  |  | 3,579 (20.7) | 1,930 (22.3) |  |  |
|  | Others | 14,108 (41.5) | 3,399 (39.2) |  |  | 6,823 (39.5) | 3,396 (39.2) |  |  |
| Education | ≤middle school | 6,139 (18.0) | 1,352 (15.6) | <0.001 | 2.998 | 2,830 (16.4) | 1,351 (15.6) | 0.002 | 1.018 |
|  | high school | 12,078 (35.5) | 3,304 (38.1) |  |  | 6,207 (35.9) | 3,301 (38.1) |  |  |
|  | ≥college degree | 15,809 (46.5) | 4,005 (46.2) |  |  | 8,256 (47.7) | 4,002 (46.2) |  |  |
| Types of housing | Detached house | 10,973 (32.2) | 3,006 (34.7) | <0.001 | 8.180 | 5,989 (34.6) | 3,000 (34.7) | 0.995 | 0.006 |
|  | Apartment | 15,208 (44.7) | 3,962 (45.7) |  |  | 7,926 (45.8) | 3,961 (45.8) |  |  |
|  | Others | 7,845 (23.1) | 1,693 (19.5) |  |  | 3,378 (19.5) | 1,693 (19.6) |  |  |
| Housing tenure type | Private | 20,335 (59.8) | 5,523 (63.8) | <0.001 | 8.700 | 11,027 (63.8) | 5,516 (63.7) | 0.684 | 0.496 |
|  | Lease | 10,442 (30.7) | 2,454 (28.3) |  |  | 4,949 (28.6) | 2,454 (28.4) |  |  |
|  | Others | 3,249 ( 9.5) | 684 ( 7.9) |  |  | 1,317 ( 7.6) | 684 ( 7.9) |  |  |

^a^*p*-values were calculated with independent sample *t*-tests or chi-squared test.
